# Sex-influenced DNA methylation differs by placental cell type

**DOI:** 10.1186/s13293-026-00869-x

**Published:** 2026-03-18

**Authors:** Jiyoung Han, Amy M. Inkster, Victor Yuan, Maria S. Peñaherrera, Wendy P. Robinson

**Affiliations:** 1 BC Children’s Hospital Research Institute, 950 W. 28th Ave, Vancouver, V5Z 4H4 BC Canada; 2 Department of Medical Genetics, University of British Columbia, Vancouver, BC Canada

**Keywords:** DNA methylation, endothelial cell, placenta, sex, X chromosome

## Abstract

**Background:**

Sex differences in the function and morphology of the human placenta can lead to sex differences in pregnancy outcomes. X chromosome inactivation (XCI) is the primary mechanism for dosage compensation between the sexes, and is strongly associated with X-chromosome promoter DNA methylation (DNAme) in somatic cells. However, in the placenta, low X-chromosome promoter DNAme has been reported. The placenta is a complex organ consisting of cells of different developmental origins, but the sex differences in DNAme by specific cell types have not been investigated.

**Methods:**

We examined sex-influenced DNAme from 18 to 19 samples each of endothelial, stromal, cytotrophoblast and Hofbauer cells, sorted from term placentas, as well as matched whole chorionic villi. We also compared these profiles with data from 65 endothelial cell samples from placental chorionic plate arteries and veins (XX = 16, XY = 13) and umbilical cord veins (XX = 22, XY = 14). All data were derived from Illumina Infinium HumanMethylation450 or EPIC DNAme arrays. Sex-stratified analyses of the X/Y and autosomal DNAme were undertaken to identify DNAme differences associated with sex chromosome complement.

**Results:**

The DNAme distribution on both the X and Y chromosomes differed by cell type. These differences clustered according to the differing developmental origins from extraembryonic mesoderm (endothelial/stromal), trophectoderm (cytotrophoblast) and epiblast (Hofbauer cells), with Hofbauer cells sharing a similar distribution with blood and umbilical endothelial cells. Interestingly, the typical XCI-associated DNAme at promoter CpG islands (CGI) on the X-chromosome of XX cells was absent for endothelial/stromal cells and present only at low levels in trophoblasts, suggesting that de novo establishment of promoter-CGI DNAme on the X-chromosome may differ by cell type.

**Conclusion:**

The lack of X-linked promoter DNAme in extraembryonic mesoderm-derived cells (endothelial/stromal) is consistent with a distinct developmental origin of these populations relative to the other placental and umbilical cell types. Autosomal DNAme also showed cell-type differences in alignment with cellular relationships observed for sex chromosomes. This work suggests the effects of sex chromosome complement on pregnancy outcomes may differ by placental cell type.

**Supplementary Information:**

The online version contains supplementary material available at 10.1186/s13293-026-00869-x.

## Highlights

- X and Y- chromosome DNA methylation differs by placental cell type.
- DNA methylation patterns are consistent with the distinct developmental origins of different placental cell types.
- DNA methylation typically present at X-linked promoters in somatic XX cells was absent in villous endothelial and stromal cells.
- Endothelial cells in the small vessels of placenta differ strikingly in DNAme from those in umbilical endothelium.

**Supplementary Information:**

The online version contains supplementary material available at 10.1186/s13293-026-00869-x.

## Abstract (summary)

The human placenta is a complex organ comprised of diverse cells of different origins, and its function and structure exhibit sex differences associated with pregnancy outcomes. We examined the DNA methylation (DNAme) of placental cells in autosomes and sex chromosomes separately to investigate the impact of sex on the DNAme of placental cells. We used 94 placental cell samples (XX = 50, XY = 44) including 18–19 samples each of endothelial, stromal, Hofbauer cells, cytotrophoblasts and whole chorionic villi, and compared these with public data from 65 endothelial cell samples derived from placental plate artery and vein (XX = 16, XY = 13) and umbilical vein (XX = 22, XY = 14). The DNAme distribution of the X-chromosome differed by cell type in a manner that reflected differing developmental origins. Female (XX) placental endothelial/stromal cells showed distinct DNAme distributions from cytotrophoblast, and both differed from that of Hofbauer cells, which shared a similar DNAme distribution with blood. Interestingly, the typical DNAme associated with X chromosome inactivation was absent or low in endothelial/stromal cells and cytotrophoblasts, suggesting that the DNAme patterns may differ by cell types and their origins. Y-chromosome and autosomal DNAme also showed cell-type differences consistent with a common developmental origin of endothelial and stromal cells distinct from other placental cell types. This work provides insight into the influence of sex and cellular developmental origin on DNAme of mature human placental cell types.

**Supplementary Information:**

The online version contains supplementary material available at 10.1186/s13293-026-00869-x.

## Background

The placenta is the core organ that mediates fetal development and growth during pregnancy. As it develops from the zygote, it normally has the same sex chromosome complement as the fetus. Sex differences in placental function may contribute to sex differential fetal development and growth [1, 2], and may be the result of several influences [3]. Differences in placental gene expression due to sex chromosome complement (XX or XY) lead to sex-influenced functional and physiological features even before the development of fetal gonads, and continue throughout gestation [4, 5]. In addition, differences in sex hormone exposure or indirect effects of fetal sex, such as differences in immune regulation, may impact placental gene expression and function [6–8].

In mammalian cells with two X chromosomes, X-chromosome inactivation (XCI) occurs to equalize gene dosage to the single X found in XY cells [9, 10]. XCI is the epigenetic silencing of one X leading to the transcription of only one copy of most X-linked genes; however, escape from XCI of some genes can lead to sex-dependent expression [11]. DNA methylation (DNAme) is one of multiple epigenetic marks acquired after the initiation of XCI [12, 13], and DNAme at promoter CpG Islands (CGI) on the inactive X chromosome (Xi) is typically viewed as a hallmark of XCI status [14, 15]. However, in the human placenta DNAme levels at X-chromosome promoters is generally lower than in fetal or adult somatic tissues [16–18]. We recently reported that there is low promoter DNAme at most genes on the inactive X chromosome in the placenta, even if they are subject to XCI [18]. We also found that cell composition affects X-chromosome DNAme profiles in the placenta [18]. However, to our knowledge, there are no studies focused on sex-influenced DNAme in the context of diverse placental cell types and their cellular origins.

Chorionic villi (CV) are the functional units of the placenta, and are comprised of cells derived from both the trophectoderm and the inner cell mass (ICM) of the blastocyst. After implantation, trophectodermal cells differentiate into mononuclear cytotrophoblasts and their fusion product, syncytiotrophoblasts, forming primary chorionic villi [19, 20]. Secondary villi are formed by migration of extraembryonic mesenchymal cells to the villous core [19, 20]. Continuous proliferation of mesenchyme and the formation of fetal capillaries leads to the development of tertiary villi [19, 20]. In addition, a subset of cytotrophoblasts penetrate the maternal decidua from the CV and are referred to as extravillous trophoblasts [21]. The villus core thus consists of ICM-derived cell types including Hofbauer cells, placental endothelial cells and stromal (fibroblast) cells. Hofbauer cells are placental macrophages which ultimately differentiate from fetal monocytes or placental erythro-myeloid progenitors [22–24].

During early development, DNAme erasure occurs in the blastocyst, and subsequent *de novo* DNAme is established in the various placental cell lineages upon their differentiation, leading to lineage specific DNAme profiles [24–26]. DNAme on autosomes tends to be lower in placenta as compared to somatic tissues, which is largely attributable to the presence of large partially methylated domains in the trophoblast [24]. DNAme associated with XCI is similarly established after implantation, as cells begin to differentiate [27], but lower DNAme of X-linked promoters in placenta, occurs independently from the global hypo-methylation associated with PMDs [18]. In addition to studying epigenetic processes like XCI, DNAme studies have demonstrated placental sex differences across the autosomes [18, 28–30], However, due to the rarity of sorted placental cell DNAme profiles, our knowledge of autosomal sex differences across the different placental cell types, and their X and Y DNAme patterns, remains unexplored.

To study the influence of sex and cell type on placental DNAme, we investigated the DNAme profiles of sex-stratified autosomes, X and Y-chromosomes of four isolated human placental cell types (endothelial, stromal, Hofbauer cells, and cytotrophoblasts) with matched whole chorionic villus samples, using the 850 K Illumina EPIC v1.0 DNA methylation array [24]. We observed extensive cell-type variation in X-chromosome DNAme, corresponding with differing cellular origins early in development. Our study shows that placental DNAme is complex, with unique sex and cell-type influenced profiles on the X and Y-chromosomes.

## Materials & methods

### Data processing

This study is based on the Illumina EPIC v1.0 methylation array data from Yuan et al. (GEO ID: GSE159526) [24]. As previously reported, all samples were karyotypically normal by multiplex ligation-dependent probe amplification. These data are derived from FACS-sorted cells from 19 term placentas (gestational age of 36.4–40.4 weeks), including trophoblast cells, Hofbauer cells, endothelial, and stromal cells, as well as matched whole chorionic villi. We are now extending our previous analysis of autosomal DNAme in these cell types to DNAme on the sex chromosomes. Analyses were performed in R version 4.3.1. The IDAT files and phenotype data were processed following our established pipeline (Supplementary Fig. 1) [31]. X and Y-chromosome probes were used to confirm the recorded sex in sample data, and 59 SNP “rs” genotyping probes, within the array, were used to identify potentially contaminated samples and possible sample mix-ups. Ancestry probabilities of whole chorionic villi samples were estimated using the PlaNET R package [32]. Sample filtering was as described in Yuan et al. and limited to the 94 term samples including 19 (XX = 10, XY = 9) chorionic villi (CV), cytotrophoblasts (CTB), endothelial (EC), stromal cells (SC) and 18 (XX = 10, XY = 8) Hofbauer cells (Supplementary Table 1).

CpG probes with fluorescence detection p-value of > 0.01 or bead count < 3 in > 5% of samples were removed, following the protocol of Inkster et al. [33] to appropriately filter the X and Y-chromosome probes. A list of cross-hybridizing probes was obtained from Zhou et al. and removed [34]. The samples used in the original Yuan et al. study were normalized by normal-exponential out-of-band (Noob) [35] and Beta-Mixture Quantile (BMIQ) normalization [36], and included the same number of samples and autosomal CpGs. On the X-chromosome, additional probes, including those in repetitive elements (n_CpGs_ = 1,975), cancer testis genes (n_CpGs_ = 622) and the X-transposed region (n_CpGs_ = 80) were removed [33]. After processing, 737,050 autosomal CpGs, 14,766 X-linked CpGs, and 293 Y-linked CpGs remained for downstream analyses.

### Cell type verification

In the original study from which the data used here derive [24] the origin of cell types was validated by (i) immunofluorescence staining of the flow cytometry sorted cells; (ii) principal component analysis; and (iii) characterizing cell type differences. The flow cytometry results of the stained and sorted cells confirmed the high purity of the sorted cells as approximately 90% for trophoblast (TB) (KRT7+, 97%), Hofbauer cells (HB) (CD68+, 95%), and placental endothelial cells (EC) (CD31+, 88%), but slightly lower purity of stromal cells (SC) (VIM+, 73%), distinguished from EC. For example, genes known to be specifically expressed in trophoblast, had regions of highly cell-specific DNAme localized near their transcription start sites. Stromal cell differentially methylated sites were enriched for intracellular processes related to maintaining tissue structure, such as “actin cytoskeleton” and “collagen binding”. Hofbauer cells were shown to cluster closely with fetal monocytes which is consistent with their origin from this population; while endothelial and stromal cells did not cluster with other cell types.

### Public endothelial cell data processing

Datasets of human placental villous arterial and venous endothelial cells (pAEs/pVEs) and human umbilical vein endothelial cells (uVEs) were used from Cvitic et al. (2018) (GSE106099) and Rhead et al. (2020) (GSE144804) in the form of IDAT files [6, 37]. Downloaded IDAT files of pAE/pVE Illumina 450 K DNAme data of 30 samples were processed by following the same processing steps above. Unnecessary autosomal probes were filtered (*n* = 476,682), and 8,830 X-linked probes were used for analyses. PCA, sample donor checks using SNPs, hierarchical clustering, and sex checks were performed to verify the sample information. The pAE sample AEC-110 was removed as it did not cluster with its reported cell type and is likely mislabeled. There was a mismatch in AEC-103 between the reported sex and the predicted sex, and sex was accordingly reassigned as “XX”. No sex was reported for the pVE sample VEC-2d but this sample could be assigned as “XY” by performing the sex check using DNAme data, leaving a total: XX = 16, XY = 13 samples available for analysis.

Downloaded IDAT files of uVE Illumina EPIC DNAme data (*n* = 74) were first filtered to remove TNF-α (Tumor Necrosis Factor alpha)-treated samples (*n* = 37) and then were processed by following the same processing steps above, including removal of 3 samples with low fluorescence intensities to exclude 38 samples in total. Samples with GEO reported sex disagreeing with DNAme-derived sex chromosome complements (*n* = 6) were relabeled according to DNAme-derived sex [33]. After processing 14,861 X-chromosome probes in 36 samples (XX = 22, XY = 14) were available for analysis. Processed 450 K and EPIC DNAme data were limited to the shared CpG probes, and a total 8,012 X-chromosome probes common to both arrays were used for data analyses.

### Annotation

Probe annotation for UCSC CpG island, location, manifest and others for Illumina 450 K and EPIC DNAme data were taken from R package IlluminaHumanMethylation450kanno.ilmn12.hg19 (v0.6.1) and IlluminaHumanMethylationEPICanno.ilm10b4.hg19 (v0.6.0) [38, 39]. The regulatory regions and UCSC annotations for the EPIC array from Bizet et al. (2022) were used for annotating CpG probes to CpG islands and regulatory regions [40].

### Differentially methylated CpGs (DMCs)

To identify differentially methylated CpGs, the R package *limma* (ver. 3.5.0) was used to build linear models with empirical Bayes moderation [41]. Linear regression models for the autosomes and X-chromosome were generated to compare the average DNA methylation between XX and XY. For Y-chromosomes, a linear model with a contrast matrix was built to compare the DNA methylation of one cell type to the average of all other cell types [24]. Statistically significant DMCs were identified at a false discovery rate (FDR) of < 0.05 and mean DNAme difference > 10%. The R package *missmethyl* (v1.36.0) was used to test the enrichment of DMCs in specific gene sets [42].

### Nucleotide BLAST analysis for Y-chromosome cross-hybridization

Command-line nucleotide BLAST was performed on the significant cell-DMCs of Y-chromosomes using the 50-nucleotide probe A and B sequences of DMCs. The blastn for short sequences (-short) was run against databases generated from the Human Genome build 19 (hg19) to filter out cross-hybridizing probes on the X chromosome. To detect any low chance of cross-hybridization on the X chromosome, sequences that match at nucleotide position 50 and are ≥ 40 bp and have ≥ 75% sequence identity to the X chromosome were excluded based on criteria from Chen et al. and Inkster et al. [28, 43].

### Motif enrichment analysis for transcription binding motifs

Local motif enrichment analysis was performed on sex-DMCs of placental endothelial cells using the CentriMo in Multiple EM for Motif Elicitation (MEME) Suite browser tool [44–46]. The sex-DMCs were examined to identify transcription factor binding motifs in the full Homo sapiens Comprehensive Model Collection (HOCOMOCO) v11, enriched for a 100 bp window centered in the input CpGs.

## Results

### X-linked DNAme variation in placental cells is driven by cell type and sex

**Fig. 1:**
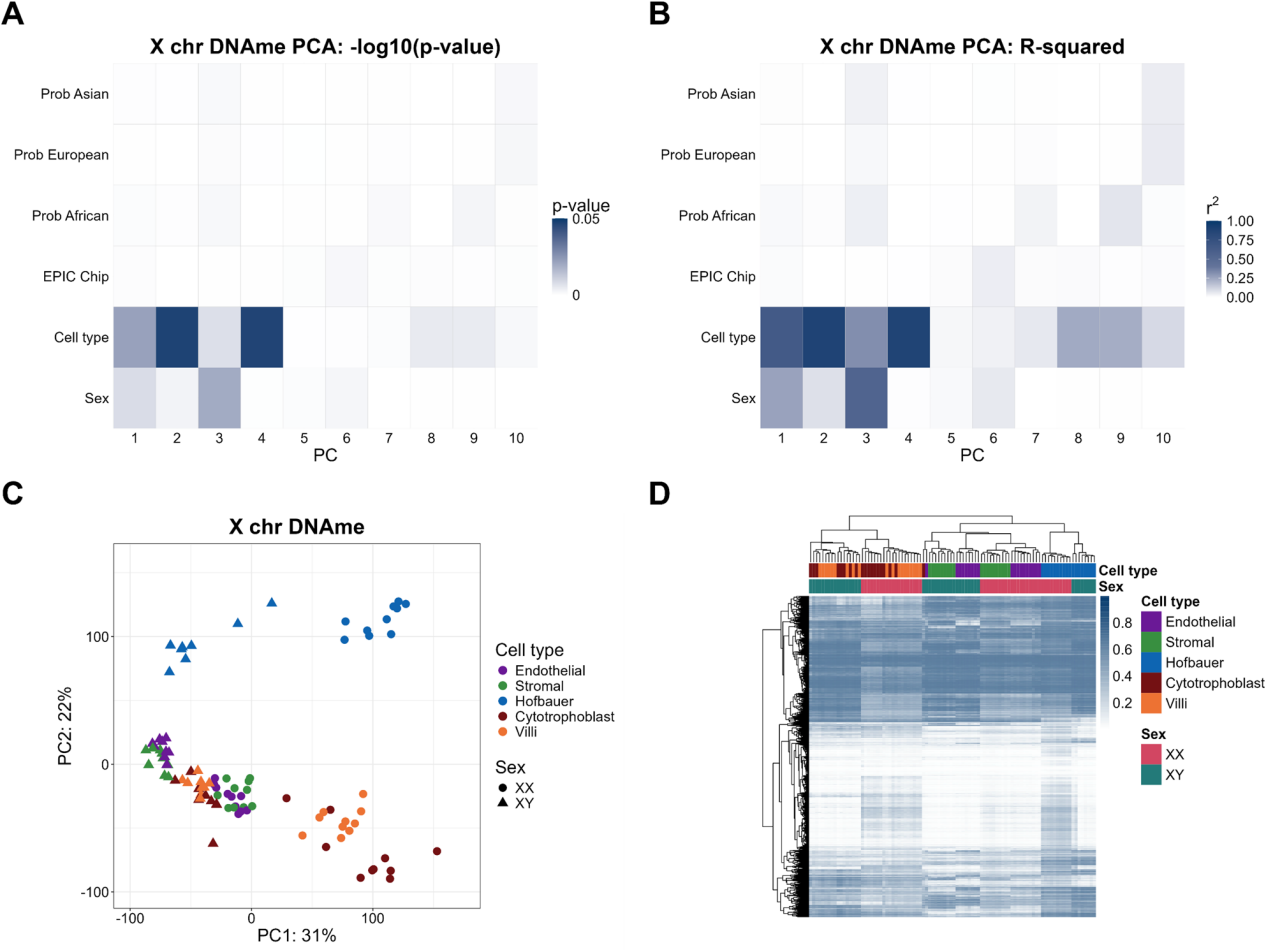
Strong influence of the sex and cell type variables in X-chromosome DNAme (n_CpGs_ = 14,766) of placental cells. (**A**) P-value of PCs versus related variables in PCA (Gradient was scaled by -log_10_ (p-value)). (**B**) R-squared p-value of PCs versus related variables in PCA. (**C**) PC1 versus PC2 scatterplots, colored by sex and cell type. (**D**) Hierarchical clustering heatmap of X-chromosome DNAme from XX and XY placental cells. Villi = whole (bulk) chorionic villi

To identify the primary variables associated with X-linked DNAme variation in the cell-type specific data from GSE159526, we first performed principal component analysis (PCA) on DNAme at 14,766 X-chromosome probes in all 94 samples. We tested for association between DNAme variation described by the principal components (PC scores) and sample variables (via linear models of the form PC ~ variables of interest) (Fig. 1A and B). Cell type of the individual DNAme sample was strongly associated with the top 4 PCs (all nominal p-values < 0.001), which together explained > 70% of the X-chromosome DNAme variation in the dataset (PC1: 30%, PC2: 23%, PC3: 15%, PC4: 6%). Sex of the sample (XX or XY) was associated with PC1 and PC3 (p-value < 0.001). Although PC1 primarily separated samples by sex (Fig. 1C), there was further subdivision by cell type within each sex along PC1, with XX stromal and endothelial cells clustering more closely to XY cells, while XX cytotrophoblasts and villi fell furthest away from XY samples along PC1. PC2 predominantly separated Hofbauer cells from all other cell types. Inferred ancestry probabilities and the technical chip variable (Sentrix ID) had only weak or non-significant associations with any of the top 10 PCs (Fig. 1A and B).

To further visualize the relationship between sex and cell type, we performed hierarchical clustering on the same 14,766 X-chromosome probes (Fig. 1D). This revealed three major clusters based on cell type: (i) cytotrophoblasts and villi, (ii) Hofbauer cells, and (iii) endothelial and stromal cells. Trophoblasts are the predominant cell type in whole chorionic villus tissue, which explains the clustering of cytotrophoblasts with villi in this analysis. Samples further subdivided by sex within each of the three main cell type clusters. Together with the PCA results, these hierarchical clustering results showed that (i) both sex and cell type are major drivers of X-chromosome DNAme in this cohort, and (ii) cell-specific patterns of DNAme are similar between cells with shared developmental origins (i.e. endothelial and stromal cells).

### The distribution of X-linked DNAme of placental cells shows three distinct patterns

**Fig. 2:**
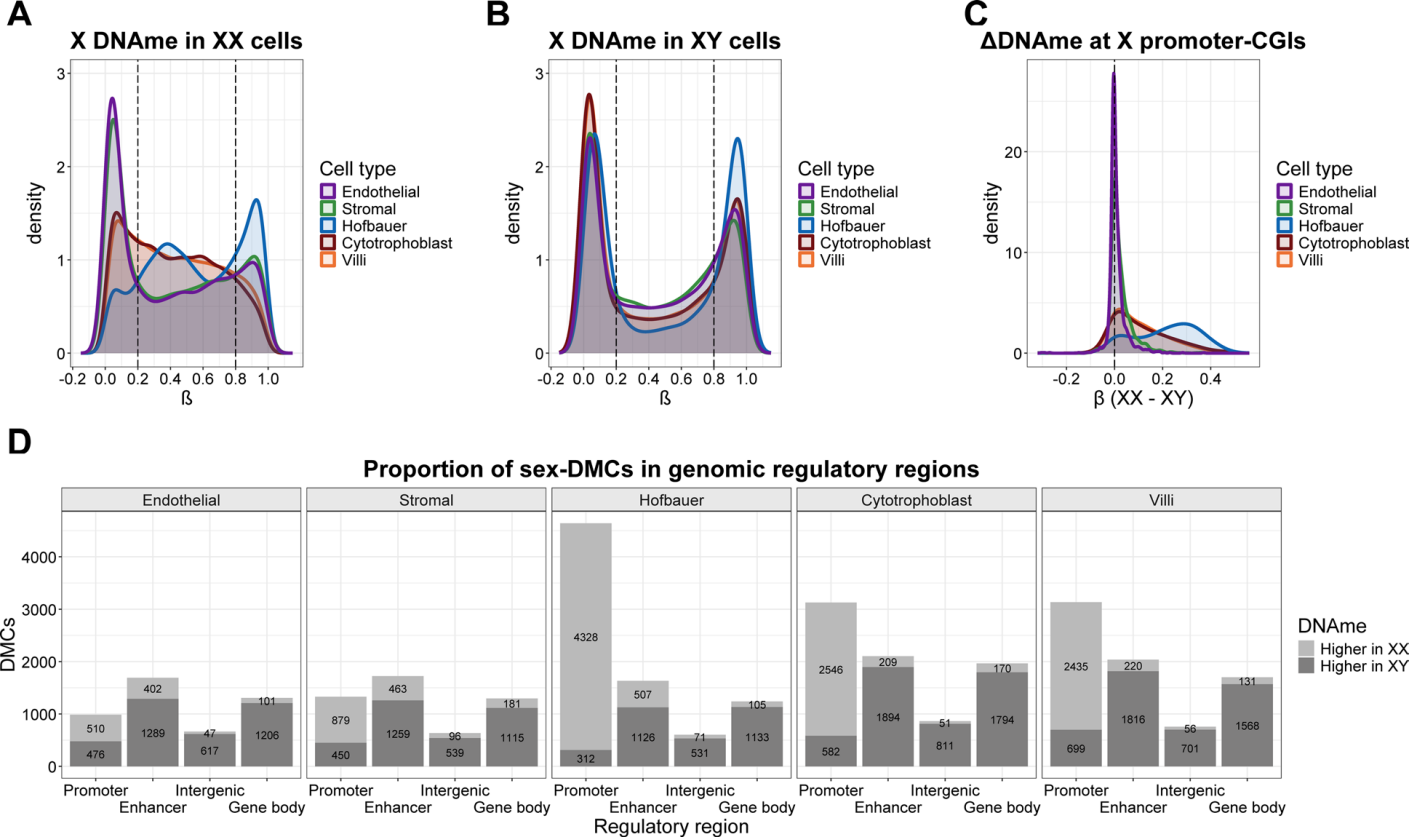
DNAme profiles of X-chromosomes in XX and XY placental cells. (**A**) Distribution of X-chromosome DNAme in XX cells at all X-linked CpGs on the EPIC array. (**B**) Distribution of X-chromosome DNAme in XY cells at all X-linked CpGs on the EPIC array. (**C**) DNAme difference between XX and XY cells at X-linked promoter CGI CpGs (Δβ = XX – XY > |0.1|). (**D**) Proportion of X-chromosome sex-DMCs (∆β > |0.1|, FDR < 0.05) in different genomic regulatory regions. Sex-DMC = sex-differentially methylated CpGs

To better understand what differentiates cell-type specific patterns of X-chromosome DNAme, we compared the distributions of X-chromosome DNAme across all cell types, separately in each sex (Fig. 2A and B, Supplementary Table 2). In XX cells, three general distributions of DNAme were observed. Hofbauer cells displayed a trimodal distribution, as is typical for somatic cells: a low methylated peak (0.2 ≤ β), a high methylated peak (β < 0.8), and a distinct peak of intermediate DNAme (0.2 < β ≤ 0.8). The intermediate DNAme peak reflects allele-specific DNAme associated with XCI, where promoters are fully methylated on the Xi and fully unmethylated on the Xa [47]. By contrast, cytotrophoblast and whole chorionic villi showed relatively few highly-methylated sites and lacked distinct intermediate methylation peaks. Both endothelial and stromal cells showed a distinct peak of low DNAme and a smaller peak of high DNAme, but had no clear intermediate DNAme peak, similar to the distribution observed amongst XY samples across all cell types.

### DNAme of X-linked promoters show few sex differences in endothelial and stromal cells

In somatic tissues, most X-chromosome gene promoters are roughly 50% methylated (β ≈ 0.5) in XX cells, and unmethylated in XY cells [48]. Although whole chorionic villi show lower DNAme at X-linked promoters relative to somatic tissues (XX), genes subject to XCI in placenta do tend to show relatively higher levels of X-linked promoter DNAme in XX versus XY samples [18]. To compare the level of X promoter DNAme amongst the different cell types we calculated the sex difference in DNAme (|∆β| = XX - XY) at the 1,393 CpGs in X-linked promoter-associated CGI, based on CGI regions defined by Bizet et al. (2022) (Fig. 2C). Sex differences in DNAme (i.e. DNAme |∆β| > 0.1) were observed at most X-linked CGIs in Hofbauer cells (76%) and at many CGI loci in cytotrophoblasts (45%). However, in endothelial and stromal cells, very few X-linked CGIs (3% and 7% respectively) had a sex difference in DNAme of |∆β| > 0.1, consistent with low/absent DNAme in both sexes at X-linked CGI promoters in endothelial and stromal cells. The lack of X-linked promoter DNAme in endothelial and stromal cells is also illustrated by a high correlation of X-linked DNAme between these cell types in both XX and XY samples (Supplementary Fig. 2A, 2B), and our PCA showing close clustering of these cell types (Fig. 1C).

In somatic cells, unlike promoters, gene bodies and intergenic regions on the X-chromosome show lower DNAme in XX compared to XY tissues [49]. We wanted to determine if the same patterns can be found in placental cells. To further evaluate DNAme by X-chromosome genomic region, we identified sex-differentially methylated CpGs (sex-DMCs) in each cell type using linear regression models with thresholds of |∆β| > |0.1| and FDR < 0.05) (Fig. 2D, Supplementary Table 3). Hofbauer cells show the pattern typically reported for somatic cells, with a high number of sex-associated DMCs (n_CpG_ = 4,640) identified in X promoter regions, most of which (93%) show XX > XY DNAme, while the majority of DMCs located in enhancer, intragenic and gene body regions show XY > XX DNAme. Interestingly, all cell types showed the expected XY > XX bias for DMCs located in enhancer, intragenic and gene body regions, with the greatest number of DMCs in these regions observed for cytotrophoblast cells. Cytotrophoblast cells also showed a predominance of XX > XY DMCs (81%) in promoters, but these occurred among a smaller total number of sex-DMCs (n_CpG_ = 3,128) as compared to Hofbauer cells, consistent with our prior finding of lower XX promoter methylation in whole chorionic villi. In contrast, stromal and endothelial cells had much lower numbers of promoter DMCs overall (n_CpG_ = 1,329 and 986), and these had nearly equal proportions of XX > XY and XY > XX DMCs (66% and 52%, respectively), indicating much less of the typical X-inactivation-associated promoter DNAme in XX stromal and endothelial cells. Given this unexpected pattern, we sought to further examine X-linked promoter DNA methylation in these two cell types.

### DNAme profile of placental endothelial cells differs from umbilical cord endothelial cells

To further characterize the unique X-linked DNAme patterns we observed in placental endothelial and stromal cells, we compared them to endothelial cells derived from other gestational tissues to determine whether we were detecting a specific endothelial X-linked DNAme signature. The endothelial cells evaluated in this work were FACS-isolated and were derived from placental microvessels within the chorionic villi. Blood flows between these placental microvessels and larger arteries and veins within the placental chorionic plate; these larger vessels then connect to the fetal vasculature via the umbilical cord. While, the placental villous endothelial cells derive from extraembryonic mesoderm, endothelial cells within the fetal compartment (fetal vessels) like the umbilical cord vessels, derive from embryonic precursors [50]. We thus sought to determine if low X-linked promoter DNAme was a shared property of all endothelial cells including those derived from the umbilical cord and large vessels of the placenta. To evaluate this, we compared DNAme patterns of our placental microvascular endothelial cells (henceforth called “pME”), to public Illumina HumanMethylation 450 K data derived from (i) cultured human placental arterial and venous endothelial cells (pAE/pVE) obtained from the chorionic plate (GSE106099) (n_pAE_ = 12, XX = 7, XY = 5 / n_pVE_ = 17, XX = 9, XY = 8), and EPIC data derived from (ii) cultured human umbilical venous endothelial cells (uVE) (GSE144804) (n_uVE_ = 36, XX = 22, XY = 14) (Supplementary Table 4).

**Fig. 3:**
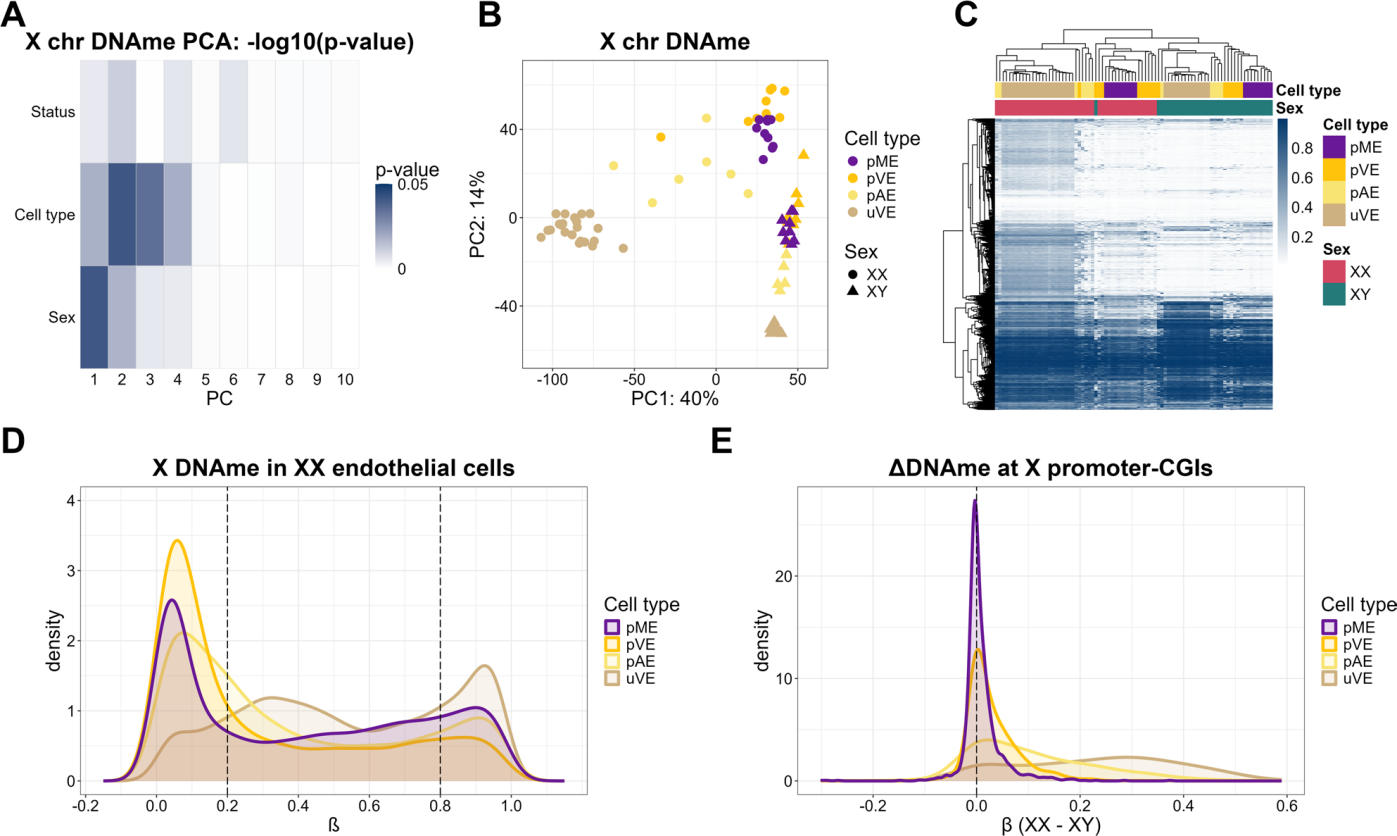
Distinctive DNAme profiles between placental and umbilical endothelial cells. (**A**) P-value of PCs versus related variables in PCA (Gradient was scaled by -log_10_ (p-value)). (**B**) PC1 versus PC2 scatterplots, colored by sex and cell type in placental and umbilical endothelial cells. (**C**) Hierarchical clustering heatmap of X-chromosome DNAme from XX and XY placental and umbilical endothelial cells. (**D**) DNAme distribution of X-chromosome from XX (pME, pAE, pVE) placental and umbilical endothelial (uVE) cells. (**E**) Promoter-CGI DNAme sex difference of placental (n_CpGs_ of pAE/pVE = 1,205, n_CpGs_ of pME = 1,388) and umbilical endothelial cells (n_CpGs_ = 1,426) and the proportion of promoter-CGIs that have low DNAme difference

PCA and hierarchical clustering on the 8,012 X-chromosome CpGs common to all endothelial cell datasets (total sample *n* = 65) demonstrated DNAme differences by both sex and endothelial cell sampling location (Fig. 3A and C). PC1 separated samples by sex and cell type, with the greatest separation between XX and XY cells observed in uVEs and least separation in pAEs (Fig. 3B). Hierarchical clustering also showed separation by both sex and cell type, although XX pVE clustered with XY samples (Fig. 3C). The X-chromosome DNAme distributions also showed similar trends (Fig. 3D), with XX uVEs showing a distinct intermediate methylated peak (57% at 0.2 < ∆β ≤ 0.8) characteristic of XX somatic cells, while in pAEs and pVEs this peak was largely absent and a large portion of X-linked CpGs had low DNAme (41%, and 57% respectively at ∆β ≤ 0.2), similar to what we observed in pMEs. In contrast, X-chromosome DNAme in XY samples was similar across all endothelial cell types.

Most promoter-CGI CpGs showed a difference in DNAme by sex (∆β > |0.1|) in uVEs (76%), but not pVEs (9%), while pAEs (44%) showed an intermediate result (Fig. 3E). We next tested the cell-type correlation in DNAme at all X-chromosome CpGs (Supplementary Fig. 3A, 3B). The strongest correlation was observed between XX pVE and pME (*r* = 0.78), and uVE and pAE (*r* = 0.75). The weakest correlation was observed between pME and uVE samples. In other words, placental microvessels (pME) studied in our first set of analyses were most similar to placental venous endothelial cells (pVE), while the umbilical cord vein (uVE) was most similar to placental artery (pAE). These results overall suggest considerable heterogeneity in the developmental origin of endothelial cells amongst these tissues, as inter-cell correlations in X DNAme between cells of similar origin is typically over 0.90 [51].

In all panels the following abbreviations are used, pME: placental microvascular endothelial cells, pAE: placental arterial endothelial cells, pVE: placental venous endothelial cells, uVE: umbilical venous endothelial cells.

### Y-chromosome DNAme varies by cell type

**Fig. 4:**
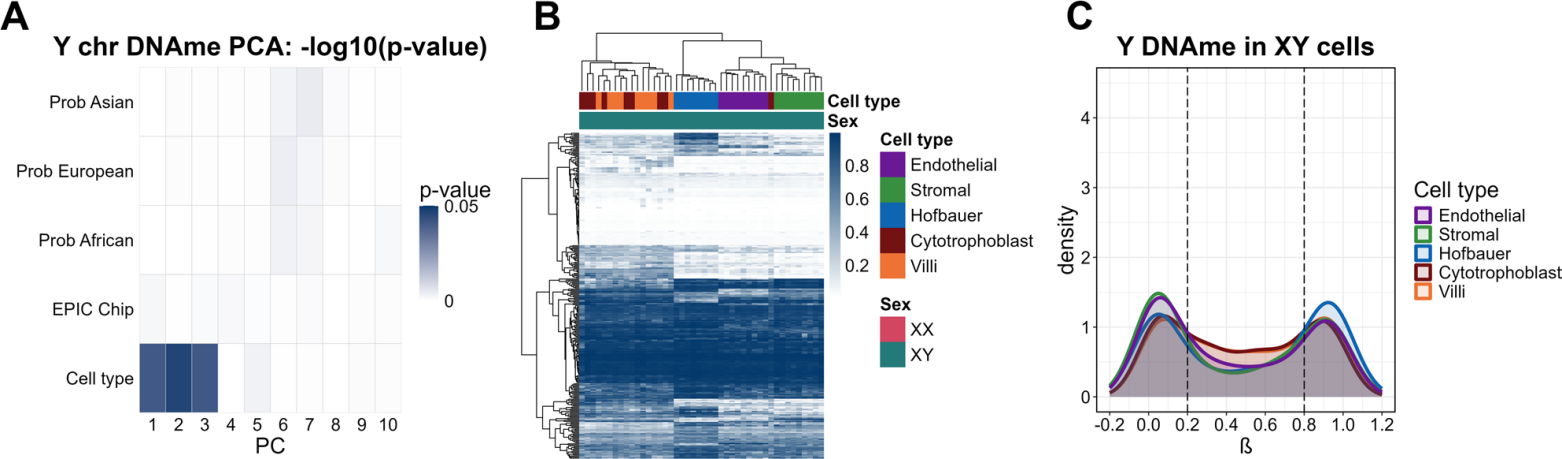
Cell-type differences in Y-chromosome DNAme (n_CpGs_ = 293). (**A**) P-value of PCs versus related variables in PCA (Gradient was scaled by -log_10_ (p-value)). (**B**) Hierarchical clustering heatmap of Y-chromosome DNAme. (**C**) DNAme distribution of Y-chromosome CpGs in XY cell types

Like the X, the Y-chromosome is also under-studied in epigenome-wide DNAme analyses. As the Y contains few genes, most of which function in the testes, we did not anticipate many DNAme differences by cell type in placenta. Nonetheless, PCA and hierarchical clustering of XY samples based on Y-chromosome DNAme (n_CpGs_ = 293) showed three distinct clusters by cell type, parallelling those observed for the X-chromosome (Fig. 4A and C). In linear models comparing the average Y-chromosome DNAme of each cell type to the average of all other cell types, we identified many cell-type influenced Y-chromosome DMCs (78, 84, 105, and 116 DMCs in endothelial, stromal, Hofbuaer cells and cytotrophoblasts, respectively) (Supplementary Fig. 4). These cell-type influenced Y DMCs overlapped 13 genes (see Supplementary Table 5). Five of these 13 genes (*DDX3Y*, *EIF1AY*, *RPS4Y1*, *USP9Y*, and *ZFY*), were previously reported to be expressed in XY term placentas [5]. These genes all possess X-linked homologs, which are also expressed in XX term placentas [5]. To confirm that Y-chromosome DNAme attributed to these X-Y homologs in our dataset was not arising from DNAme array probes cross-hybridization to their X-linked pairs, we performed a Command-line nucleotide BLAST (blastn) for short sequences on the 50-nucleotide probe sequences (probeSeqA/B) of significant Y-linked cell-DMCs of all cell types to exclude any possibility of cross-hybridization on the X chromosome due to its sequence similarity in the pseudo-autosomal region of the X and Y chromosomes. None of the Y DMC probes filtered by the selected criteria matched, suggesting that our results mostly reflect true Y-chromosome DNA methylation patterns.

### Sex-influenced autosomal DNA methylation differs by cell type

**Fig. 5:**
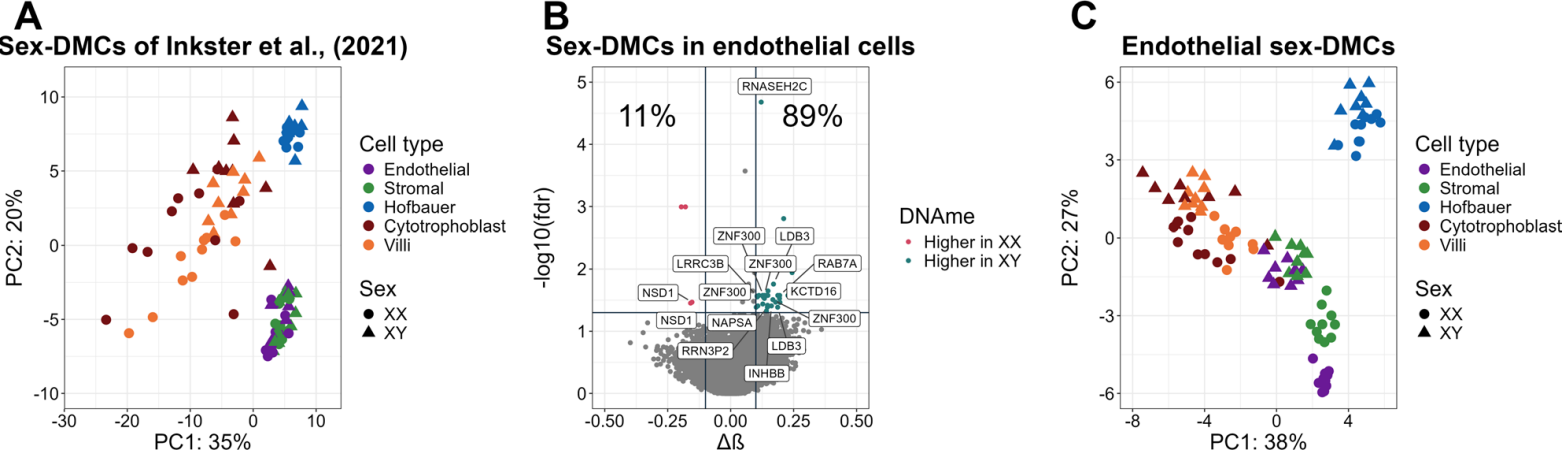
Sex-influenced DNAme of autosomes. (A) PC1 versus PC2 scatterplot of placental cells using the 145 sex-DMPs from Inkster et al. [3] colored by sex and cell type. (**B**) Volcano plot of sex-influenced differentially methylated CpGs (DMCs) in endothelial cells. (**C**) PC1 versus PC2 scatterplot of 4 types of placental cells and whole chorionic villi for the 35 significant endothelial sex-influenced DMCs colored by sex and cell type

We previously reported 145 CpGs on the autosomes that show sex-influenced DNAme in whole chorionic villi [28]. To determine if these sex-influenced autosomal CpGs were consistent across placental cell types we performed PCA using these 145 sex-influenced CpGs in the sorted placental cells. In scatterplots of PC1 versus PC2, samples predominantly separated by cell type and not sex; only the cytotrophoblasts/chorionic villi separated by sex (Fig. 5A). These results likely reflect that the 145 CpGs were originally identified as having sex differences in data from bulk villi, and may not show a sex difference in other cell types.

Although our samples sizes were small, we wanted to evaluate the existence of sex-influenced autosomal DNAme in the individual placental cell types. By comparing DNAme in each cell type by sex using linear models, we identified multiple significant sex-influenced DMCs in endothelial cells (n_CpGs_ = 35 DMCs at FDR < 0.05 and Δβ > |0.1|) and whole chorionic villi (n_CpGs_ = 7 DMCs at FDR < 0.05 and Δβ > |0.1|) (Fig. 5B, Supplementary Fig. 5), but none in the other cell types. Of the 35 endothelial sex-associated DMCs, the majority (89%) had higher DNAme in XY as compared to XX cells, and were in promoter or enhancer regions. Among the endothelial-associated sex-DMCs were CpGs in genes including *LDB3*, *INHBB*, *NSD1*, *RAB7A*, and *ZNF300*; of these genes *LDB3* and *ZNF300* had 2 or more DMCs each (n_CpGs_ = 2/4, respectively) that were also identified as differentially methylated regions (DMRs) using DMRcate R package. Previously, Inkster et al., [3] found no enrichment for estrogen or androgen receptor response elements, but for several transcription factors amongst the autosomal sex-associated DMCs. The replicated motif analysis found a significant binding motif for CXXC1 identified within 200 bps of the endothelial cells’ DMCs (adjusted P value < 0.05 and CentriMo E-value < 1), although we are underpowered give the limited number of significant hits (n_CpGs_ = 35). As we were likely underpowered to detect sex differences in all cell types, to evaluate whether the sex differences in DNAme identified in endothelial cells were truly cell-specific, we performed PCA on the endothelial sex-associated DMCs in all placental cell types. The scatterplot of PC1 versus PC2 (Fig. 5C) showed separation by cell type on PC1, with separation by sex observed in all cell types along PC2. Not surprisingly, the sex difference in endothelial cells was greatest, however, these results suggest that some of the identified DMCs show sex differences across multiple cell types, and that we are likely underpowered and missing significance in detecting sex-associated DMCs in other sorted placental cell types.

Finally, considering sex differences in DNAme at specific genes, the *ZNF300* gene was previously reported to be differentially methylated by sex in Inkster et al., [3], and was shown to be associated with placental morphology and development [52]. In endothelial cells, we identified 4 sex-DMCs at *ZNF300* (DNAme XY > XX in all cell types except Hofbauer cells), in the same promoter region reported to be sex-DMCs in Inkster et al., (2019). Among the other sex-DMCs we identified in endothelial cells, several were associated with *NSD1* and *LDB3*: 2 DMCs were observed in *NSD1* (Nuclear Receptor Binding SET Domain Protein 1) and *LDB3* (LIM domain binding 3), which plays a role as histone lysine methyltransferase and generate proteins maintaining the stability of the muscle structure, with higher DNAme in XX than XY cells (both endothelial and cytotrophoblast). Figures exemplifying the sex-differential DNAme patterns at the promoter regions of these genes are shown in Supplementary Fig. 6.

## Discussion

**Fig. 6:**
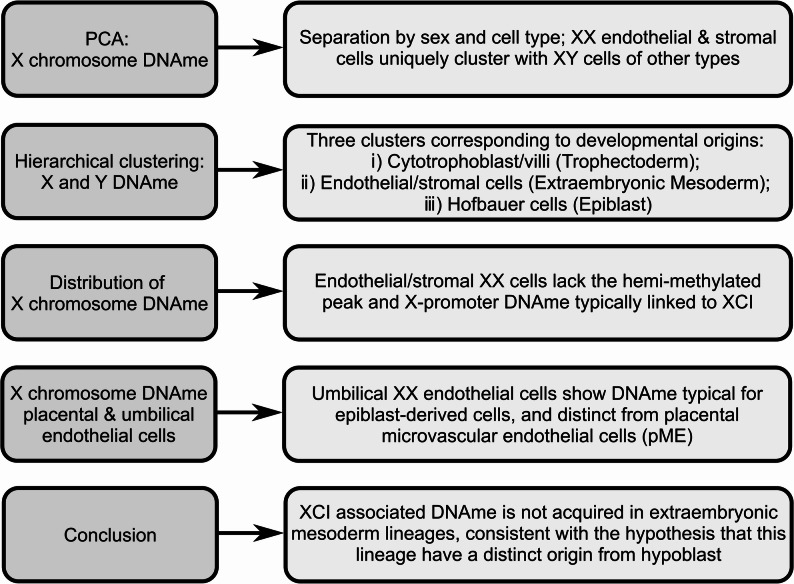
Summary of evidence for a distinct origin of placental endothelial and stromal cells as characterized by sex-stratified DNAme

In this study, we characterized patterns of X and Y-chromosome DNAme and sex-influenced autosomal DNAme in the major placental cell types (endothelial, stromal, Hofbauer, and cytotrophoblast cells), and whole chorionic villi. Specifically, we observed that placental cell types occupy one of three broad patterns of X-chromosome DNAme: (i) Endothelial/Stromal, (ii) Hofbauer, and (iii) Cytotrophoblast/Trophoblast, suggesting that patterns of X-linked DNAme may differ by the developmental origins of placental cells. We observed a similar three-group pattern of cell-type DNAme differences on the Y-chromosome, further supporting that these DNAme groups likely represent distinct developmental lineages (Fig. 6).

DNAme, including that on the X-chromosome, is largely erased in the first few cell divisions after fertilization, with *de novo* DNAme occurring near or after blastocyst implantation. XCI is characterized by the accumulation of epigenetic marks on one of the two X-chromosomes in XX cells, and is associated with gene silencing on the inactive X-chromosome (Xi). In the early implantation period, XCI occurs as tissues differentiate from the blastocyst in humans [53, 54]. In somatic cells, CGI promoters are typically highly methylated on the Xi when the associated genes are silenced [15, 47]. In contrast, human placental chorionic villus samples show low levels of DNAme of promoters on the Xi relative to all somatic tissues studied, however allelic inactivation at the level of gene expression does still occur [55] and is somewhat correlated with DNAme levels [18].

Here we show that the distribution of DNAme on the X in cytotrophoblast cells mirrors patterns observed in whole chorionic villi with low, but not absent, promoter DNAme in XX samples. Cytotrophoblast cells are derived from the trophectoderm and together with their fusion product, the multinucleated syncytiotrophoblast, are the primary components of chorionic villi, typically accounting for > 80% of DNA content in bulk whole villi tissue [24, 31]. These cells have previously been reported to have similar autosomal DNAme profiles to whole chorionic villi, as well as syncytiotrophoblast [24]. We now extend those observations to the sex chromosomes.

Hofbauer cells are a minor cell population making up < 5% of cells in whole villi [24]. We observed that the X-chromosome DNAme distribution of Hofbauer cells resembled the pattern found in XX somatic cells, including blood and buccal cells, with large peaks of intermediate promoter DNAme reflecting methylation on the Xi and lack of DNAme on the Xa [47, 56]. The developmental origin of Hofbauer cells has been debated, and may vary with both the time of gestation and potentially the cell type isolation approach. It was proposed that mesenchymal progenitor cells give rise to first trimester Hofbauer cells [57, 58], while second and third trimester Hofbauer cells are proposed to derive from fetal monocytes [59, 60]. It has also been suggested that hypoblast-derived placental erythro-myeloid progenitors differentiate into Hofbauer cells throughout pregnancy [61]. Others have also suggested Hofbauer cells in term placentas have an epiblast origin [62, 63]. In our previous investigation of autosomal DNAme from this same dataset Hofbauer cells lacked many placenta-specific DNAme features such as partially methylated domains and placenta-specific imprinting, and in addition, hierarchical clustering of DNAme of Hofbauer cells with cord blood cell types showed Hofbauer cells clustered closest to monocytes, consistent with an origin from this cell type [24].

Although XX trophoblast cells have low mean X-linked promoter DNAme, we observed that endothelial and stromal cells had lower still X-linked promoter DNAme. During XCI, multiple epigenetic marks accumulate on the inactive X, of which DNAme is the last and is hypothesized to be important in XCI maintenance [64, 65]. In whole villi, which reflects largely trophoblast, XCI silencing appears to occur to a similar level as in somatic cells [55], despite low DNAme of X-linked promoters, suggesting that DNAme is not essential for XCI maintenance in these cells [18]. The same may be true in endothelial and stromal cells, however, the largely absent promoter DNAme could indicate a greater potential for XCI escape in these cells. Previous work has shown that under some circumstances cultured chorionic villi, which represent mainly placental fibroblasts (stroma), can undergo reactivation of certain X-linked genes after depletion of DNAme via treatment with 5-deazacytidine [66], suggesting a potential functional importance of our results. Additional evidence toward potential instability of XCI in stroma comes from hybrids of cultured human term chorionic villi (fibroblasts) hybridized with mouse cells, which were demonstrated to reactivate a subset of X-linked genes [66].

Placental endothelial and stromal cells derive from extraembryonic mesoderm (exM) [67], the developmental origins of which (in humans) are still uncertain. While the literature commonly cites these cells as being epiblast-derived, this seems largely inferred from animal models (i.e. mouse, rats), and may not be comparable to the human cells examined here, given the lack of villus structure in rodent placentas [68, 69]. Studies in rhesus monkey and in vitro exM models have proposed a hypoblast (primitive endoderm) origin of primate extraembryonic mesoderm [70–73]. Our previous studies of autosomal DNAme also demonstrated that endothelial and stromal cells have similar DNAme profiles to each other, and showed intermediate DNAme patterns relative to trophoblast and Hofbauer cells with regard to partially methylated domains and placental-specific imprinted regions. Our X-chromosome DNAme results support an origin from hypoblast or very early epiblast, as the endothelial and stromal cell sex chromosome DNAme patterns are distinct from both Hofbauer and trophoblast cells, without being intermediate between the two. Intriguingly, in the post-implantation blastocyst, the acquisition of *de novo* DNAme was much slower in hypoblast and reached lower ultimate levels, as compared to the trophectoderm and epiblast [54], consistent with a distinct timing of epigenetic programming in cells derived from this origin. However, it should be noted that we previously found that partially methylated domains (PMDs) are more distinct (lower methylated) in trophoblast cells as compared to other cell types [24]. We also reported that the hypomethylation of X-linked promoters in placenta, was not linked to associated with PMDs [18]. The present data similarly suggests that the acquisition of DNAme on the X chromosome in XX cells is likely unlinked to general hypomethylation of placental tissue which has been attributed to PMDs.

**Fig. 7:**
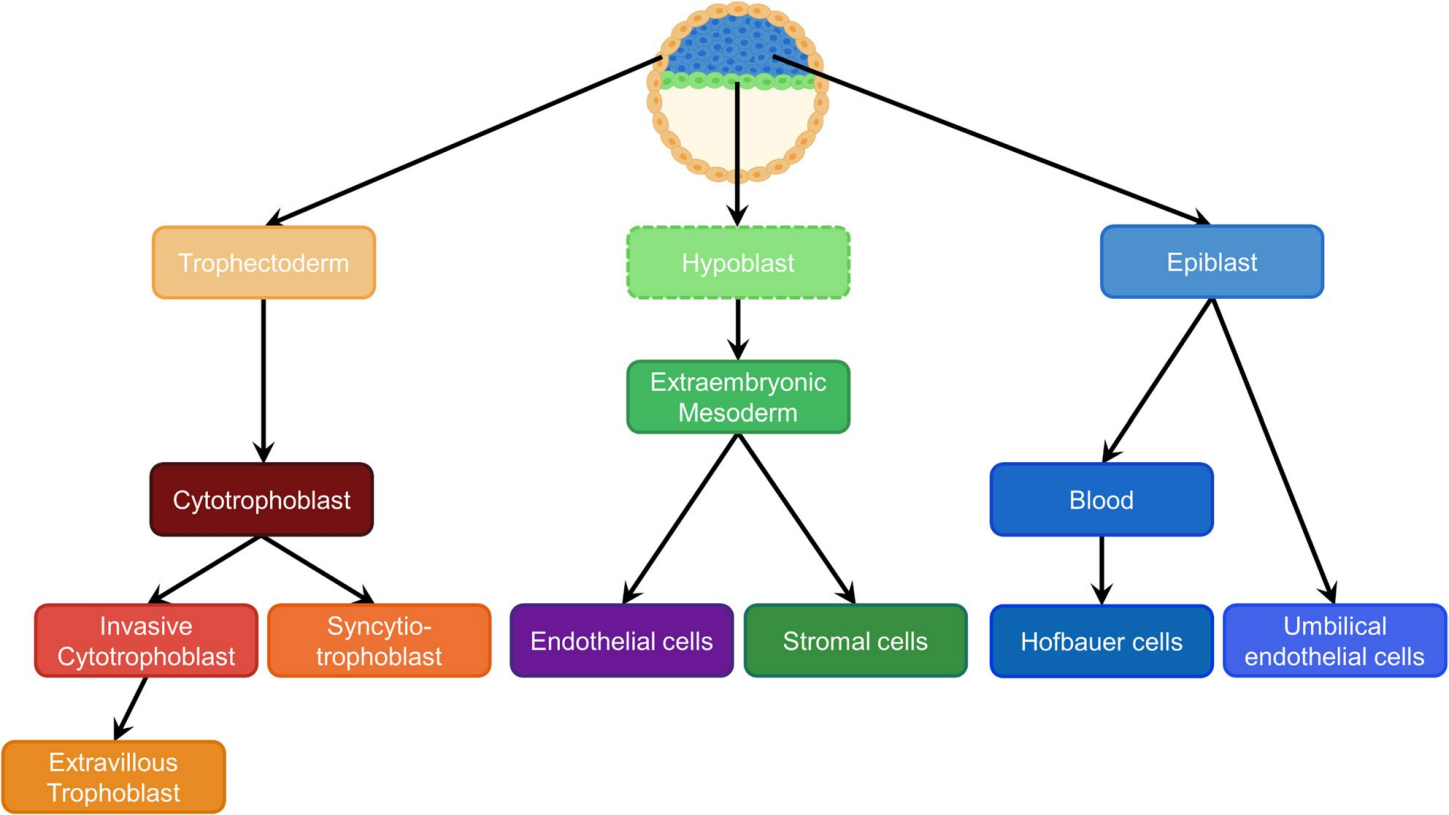
Proposed Cellular developmental origins and lineages of the cells found in the placenta

To further understand the developmental origin of placental-derived endothelial cells, we compared our data from pMEs to publicly available DNAme data derived from larger placental and umbilical vessels: pAEs, pVEs and uVEs. The pME data derive from endothelial cells isolated by FACS from microvessels within the terminal chorionic villus, while the pAE and pVE were collected from the large vessels in the placental chorionic plate which extend into the primary and intermediate chorionic villi [74]. Placental microvascular endothelial cells (pME) are derived from extraembryonic mesoderm from 18 to 20 days post-conception and before the connection to a fetal umbilical cord [67, 75]. In contrast, the umbilical venous endothelial cells (uVE) are macrovascular cells from umbilical cord [6], which connects to the fetal vessels deriving from embryonic mesoderm (epiblast origin) [74, 76, 77]. Although these pAE, pME, pVE, and uVE vessels are physically connected, there is likely a transition zone with a gradient of cells from extraembryonic to embryonic origins within the placental macro-vessels (Supplementary Fig. 7). As we expected, uVEs had X-chromosome DNAme patterns consistent with their embryonic origin, and strikingly different from the low DNAme seen in pMEs, while the pAEs/pVEs showed an intermediate pattern likely reflecting the presence of mixed cells of embryonic and extraembryonic origins in pAE [78]. We cannot definitively exclude that the differences in the experimental design in addition to the cell compositions of the various datasets might have contributed to the DNAme differences observed in these analyses. Our proposed developmental scheme based on this data is shown in Fig. 7.

As sex differences in autosomal DNAme have been reproducibly observed in whole chorionic villi, we investigated how it differs by placental cell types. Some sex-DMCs were identified in endothelial cells and some of which were shared, while others differed by cell type. For example, *ZNF300*, showed similar sex differences across cell types except for the Hofbauer cells, as also reported by Andrews et al. [29] using the same placental cell data. Whereas *NSD1* and *LDB3* had limited DNAme sex differences only in specific cell types.

We acknowledge that our results have limitations. First, our study utilized data from previously published placental cell types isolated using FACS, which may not fully represent all cells in the placenta. Second, our sample size per cell type was small, increasing variability and limiting our ability to detect subtle DNAme differences. The Illumina microarrays are also underrepresented for probes on the X and Y chromosome. This made it difficult to fully evaluate how DNAme may differ by cell type on the Y chromosome especially, although the cell-type differences observed are intriguing and support what was observed in other genomic regions. Further, we do not have matched gene expression data from these cells to determine the relationship between DNAme and X-linked gene expression, in particular whether there is more escape from XCI in endothelial/stromal cells as compared to other cell types and somatic tissues. Finally, with our current data, we cannot distinguish DNAme arising from the active and inactive X-chromosomes of XX cells and results represent an average of these distinct DNAme environments.

## Conclusions

This study provides a comprehensive characterization of sex and cell-type influenced DNAme in the placenta. Our results suggest that X-linked DNAme differs in ways that correspond to early origins of major cell type compartments of the mature placenta. Finally, our analyses add evidence that mesenchymal cells, including endothelial and stromal cells, may originate from hypoblast derived exM in the human placenta.

## Supplementary Information

Supplementary Material 1.

Supplementary Material 2.

## Data Availability

No novel datasets were generated during the current study. The datasets used include: GSE159526; GSE106099; and GSE144804.
