# Supplementary material for: Sex-influenced DNA methylation differs by placental cell type
Source: Biol Sex Differ. 2026 Mar 18;17:98. doi: 10.1186/s13293-026-00869-x (PMC13147677; doi:10.1186/s13293-026-00869-x)
Supplement: Supplementary file 2 — Supplementary Material 2. [file 13293_2026_869_MOESM2_ESM.docx]

**Supplementary Figures**


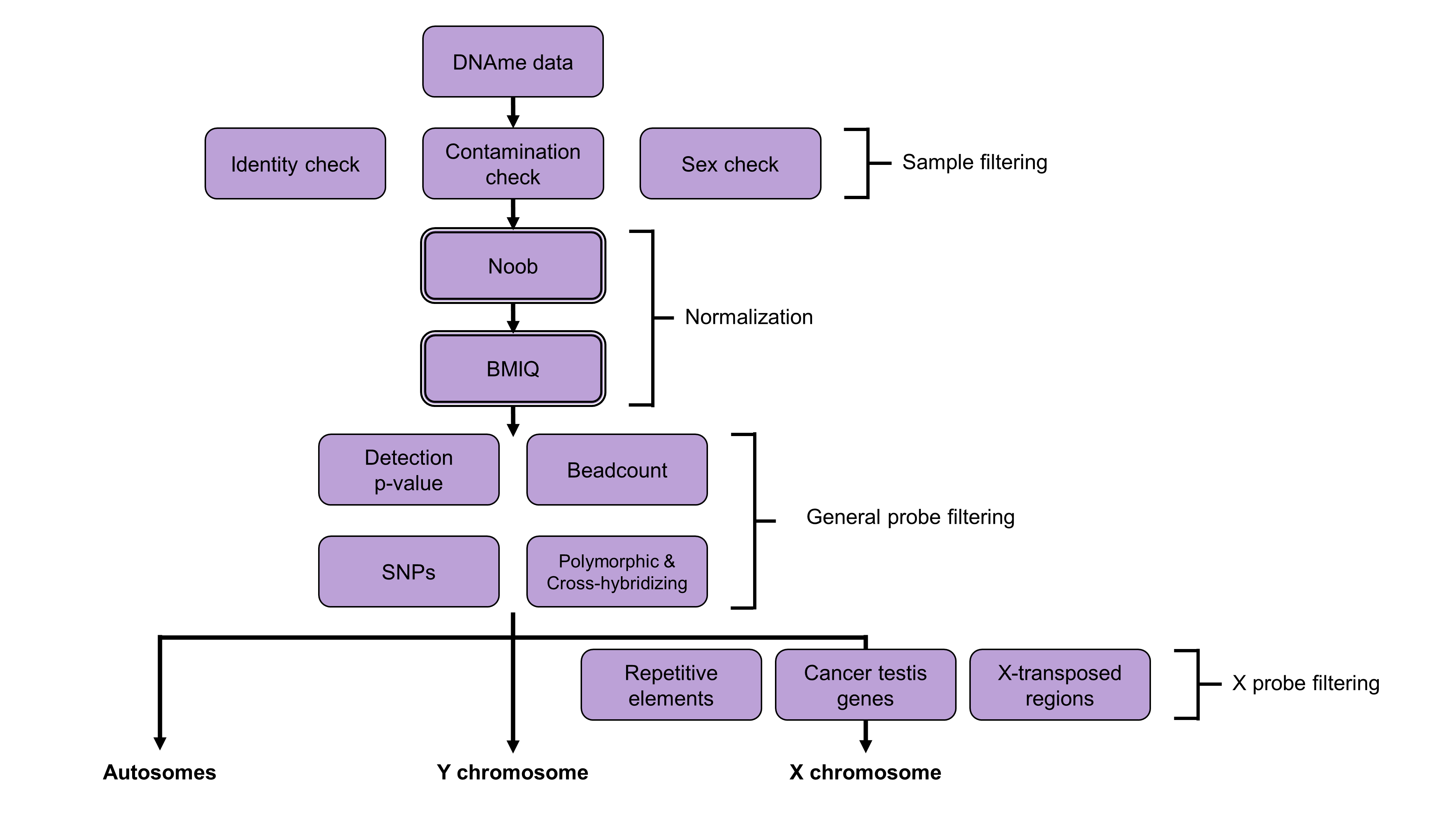
 **Supplementary figure 1. Illumina DNAme data QC and processing for autosomes and X/Y.**


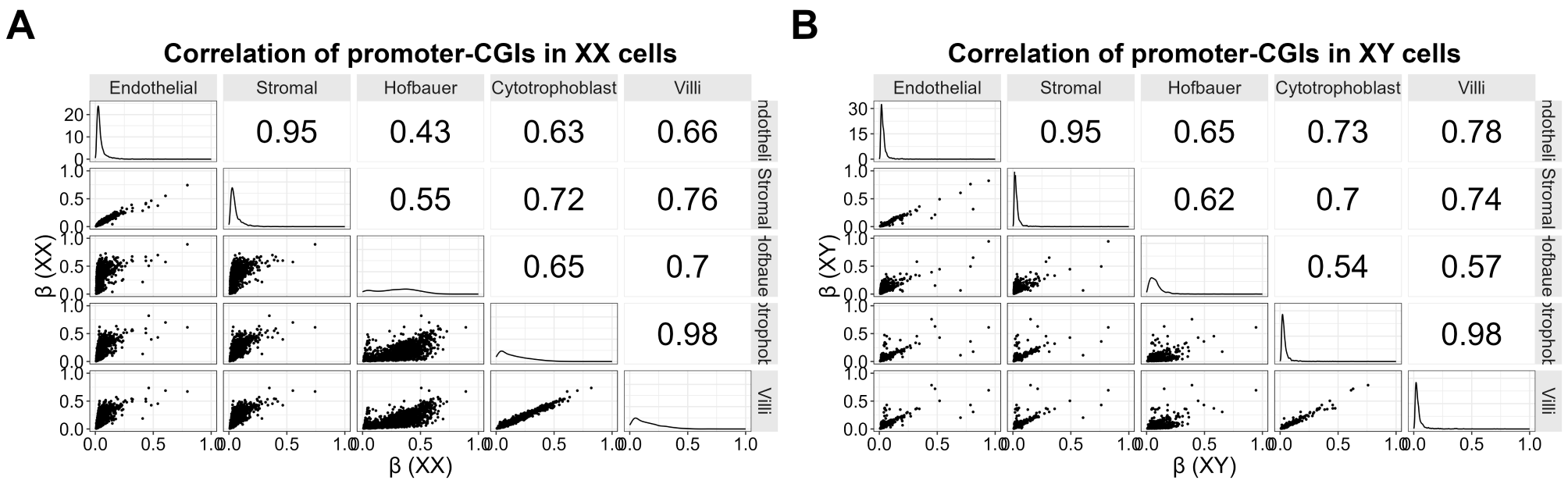


**Supplementary figure 2. DNAme correlation of the X-chromosome in XX and XY placental cells.** (A) Correlation of promoter-CGIs in XX cells. (B) Correlation of promoter-CGIs in XY cells.


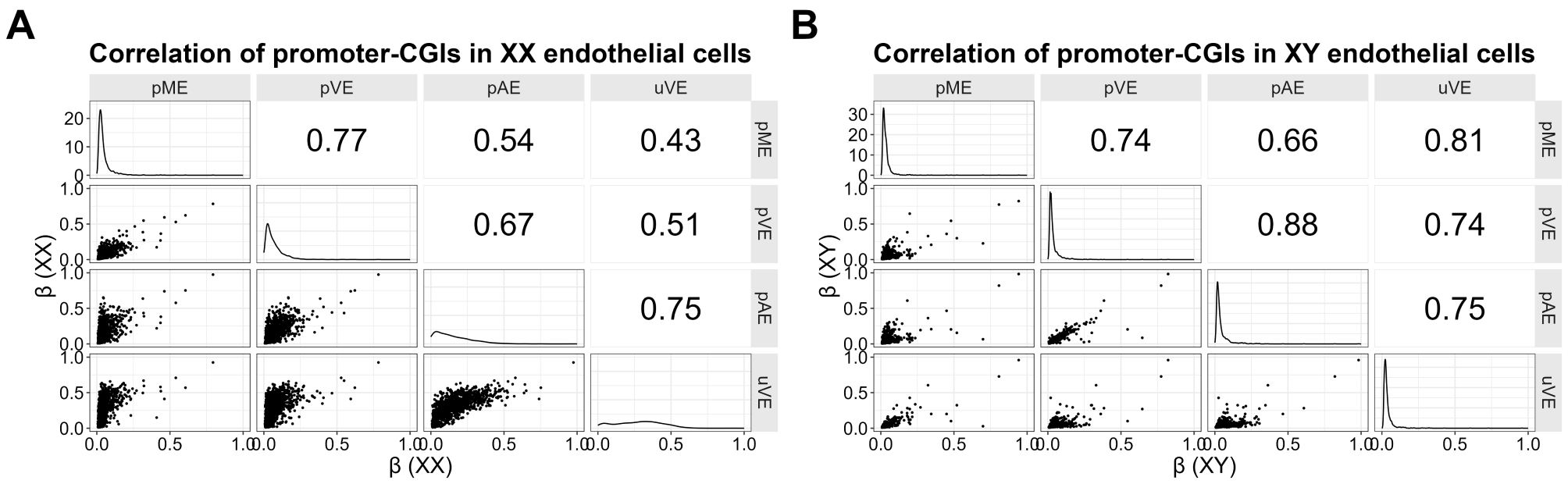


**Supplementary figure 3. DNAme correlation of the X-chromosome in XX and XY placental and umbilical endothelial cells.** (A) Correlation of promoter-CGIs in XX cells. (B) Correlation of promoter-CGIs in XY cells.


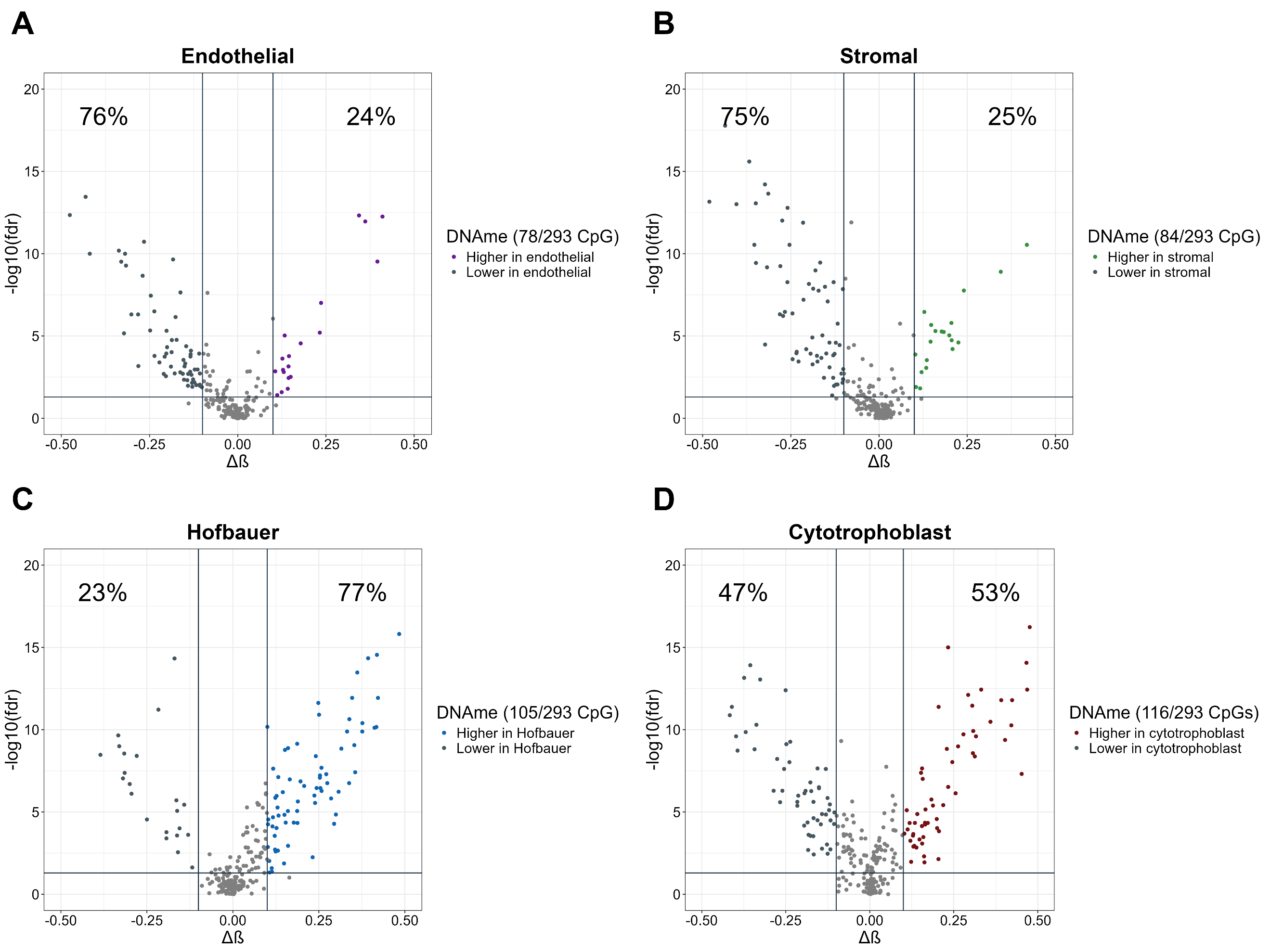


**Supplementary figure 4. Volcano plots of cell-type influenced differentially methylated CpGs (DMCs) in the Y-chromosome showing higher and lower DNAme in a given cell type versus the average of other cell types.** (A) Cell-type influenced DMCs of endothelial cells in the Y-chromosome (n_CpGs_ = 78). (B) Cell-type influenced DMCs of stromal cells in the Y-chromosome (n_CpGs_ = 84). (C) Cell-type influenced DMCs of the Hofbauer cells in the Y-chromosome (n_CpGs_ = 105). (D) Cell-type influenced DMCs of cytotrophoblast in the Y-chromosome (n_CpGs_ = 116).


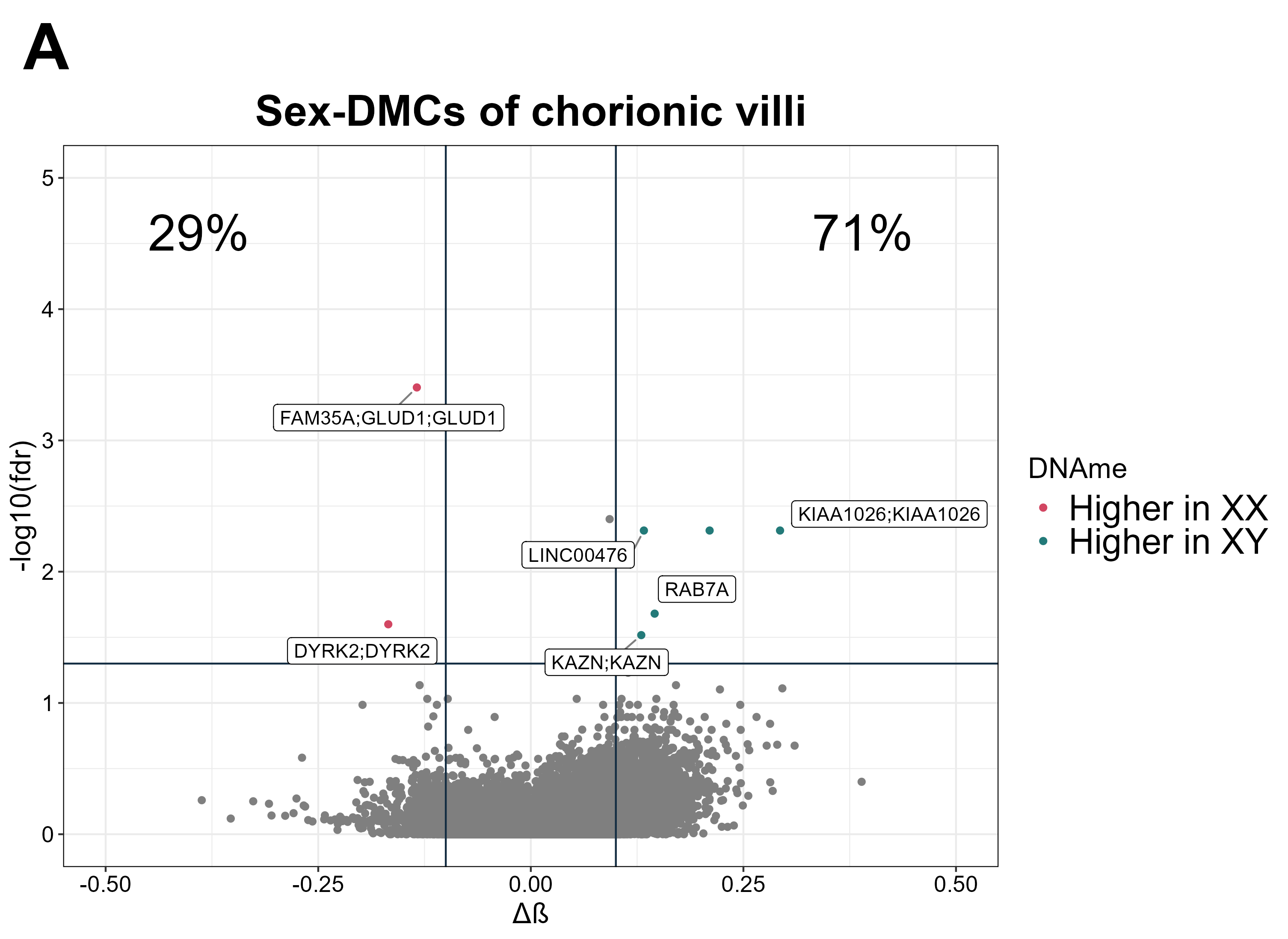


**Supplementary figure 5. Volcano plots of sex-influenced differentially methylated autosomal CpGs (DMCs) in whole chorionic villi.** (A) Sex-DMCs of chorionic villi.


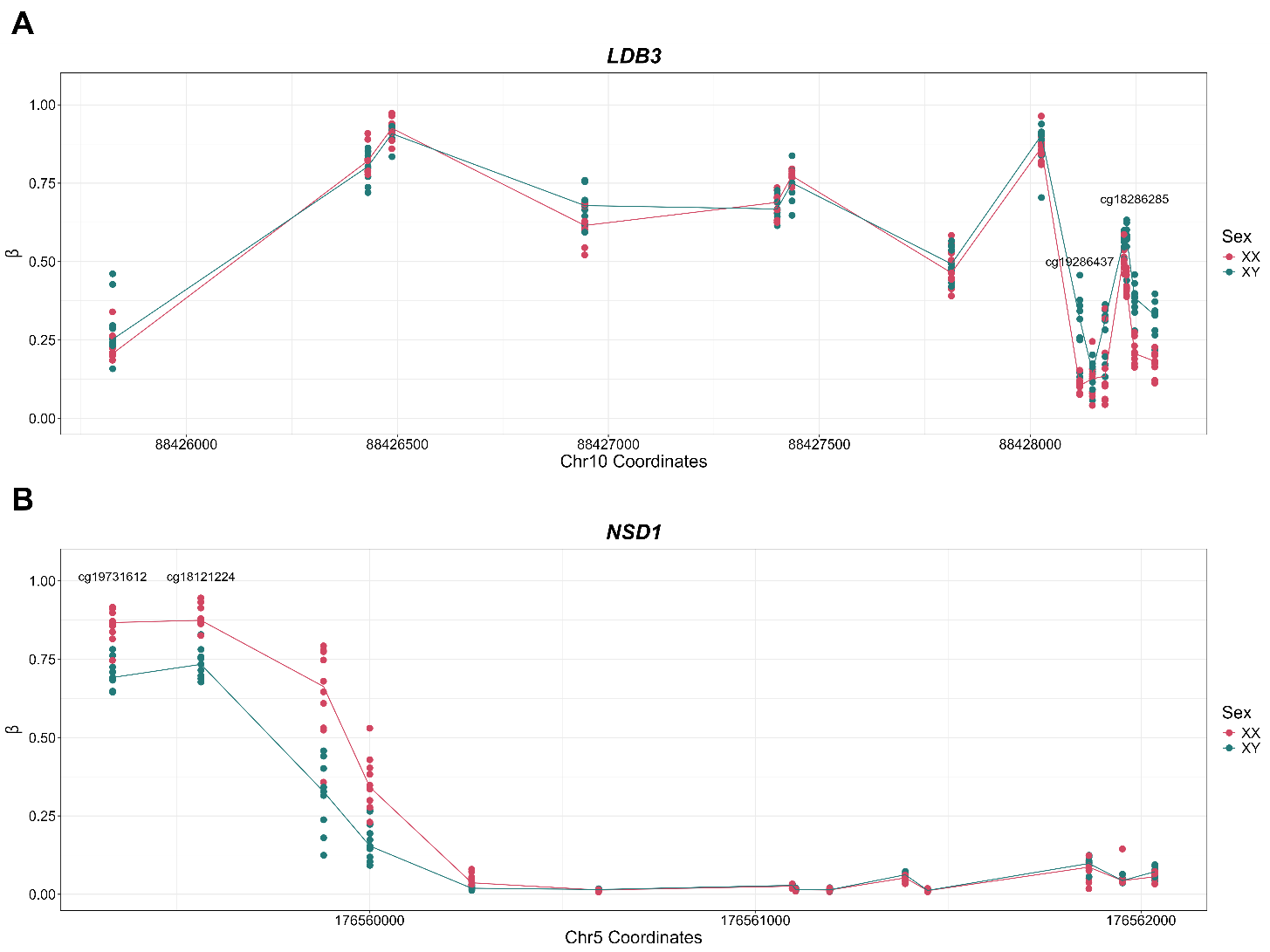


**Supplementary figure 6. Average DNAme of placental endothelial cells in *LDB3* and *NSD1* promoter.** The CpGs in the promoter were visualized and selected by using the R shiny app *Placental Methylome Browser* (https://wvictor.shinyapps.io/dmr-project/) following the “promoter” annotation. (A) Average DNAme of the CpGs in the promoter of *LDB3* (n_CpG_ = 16). The two significant sex-DMCs were labelled with their probeID. (B) Average DNAme of the CpGs in the promoter of *NSD1* (n_CpG_ = 14). The two significant DMCs were labelled with their probeID.


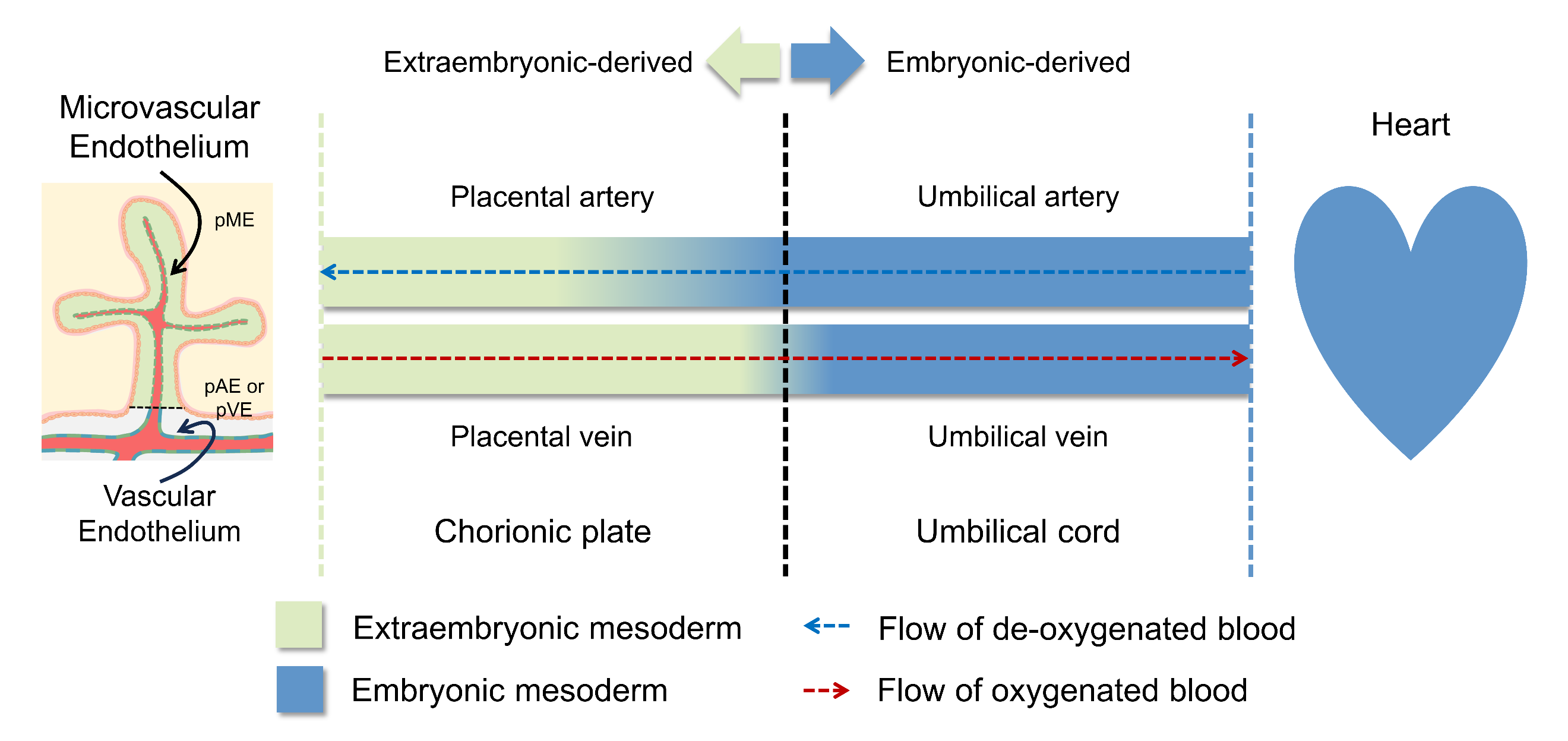


**Supplementary figure 7. Expected projection of embryonic and extraembryonic origin derived vessels.**
